# Analysis of Surgical Site Infection after Musculoskeletal Tumor Surgery: Risk Assessment Using a New Scoring System

**DOI:** 10.1155/2014/645496

**Published:** 2014-01-08

**Authors:** Satoshi Nagano, Masahiro Yokouchi, Takao Setoguchi, Hiromi Sasaki, Hirofumi Shimada, Ichiro Kawamura, Yasuhiro Ishidou, Junichi Kamizono, Takuya Yamamoto, Hideki Kawamura, Setsuro Komiya

**Affiliations:** ^1^Department of Orthopaedic Surgery, Graduate School of Medical and Dental Sciences, Kagoshima University, 8-35-1 Sakuragaoka, Kagoshima City, Kagoshima 890-8520, Japan; ^2^The Near-Future Locomotor Organ Medicine Creation Course (Kusunoki Kai), Graduate School of Medical and Dental Sciences, Kagoshima University, 8-35-1 Sakuragaoka, Kagoshima City, Kagoshima 890-8520, Japan; ^3^Department of Medical Joint Materials, Graduate School of Medical and Dental Sciences, Kagoshima University, 8-35-1 Sakuragaoka, Kagoshima City, Kagoshima 890-8520, Japan; ^4^Infection Control Team, Kagoshima University Hospital, 8-35-1 Sakuragaoka, Kagoshima City, Kagoshima 890-8520, Japan

## Abstract

Surgical site infection (SSI) has not been extensively studied in musculoskeletal tumors (MST) owing to the rarity of the disease. We analyzed incidence and risk factors of SSI in MST. SSI incidence was evaluated in consecutive 457 MST cases (benign, 310 cases and malignant, 147 cases) treated at our institution. A detailed analysis of the clinical background of the patients, pre- and postoperative hematological data, and other factors that might be associated with SSI incidence was performed for malignant MST cases. SSI occurred in 0.32% and 12.2% of benign and malignant MST cases, respectively. The duration of the surgery (*P* = 0.0002) and intraoperative blood loss (*P* = 0.0005) was significantly more in the SSI group than in the non-SSI group. We established the musculoskeletal oncological surgery invasiveness (MOSI) index by combining 4 risk factors (blood loss, operation duration, preoperative chemotherapy, and the use of artificial materials). The MOSI index (0–4 points) score significantly correlated with the risk of SSI, as demonstrated by an SSI incidence of 38.5% in the group with a high score (3-4 points). The MOSI index score and laboratory data at 1 week after surgery could facilitate risk evaluation and prompt diagnosis of SSI.

## 1. Introduction

Surgical site infection (SSI) is defined as an infection at the site of direct operative manipulation that develops within 30 days of operation if no artificial materials (implants) are used or within 1 year if artificial materials are used [1]. In general, the incidence of SSI following orthopaedic surgery has been reported to be 1% to 3% [2]. The incidence of SSI following orthopaedic surgery in Japan is 0.83% for cases of spinal canal stenosis, 0.28% for cases of disc herniation, 0.80% for cases of total hip arthroplasty (THA), and 0.96% for cases of total knee arthroplasty (TKA) [3]. This indicates that the incidence of SSI following surgery with the use of artificial materials is higher than that in cases without the use of artificial materials.

The incidence of SSI following surgical treatment for cancer is relatively high, with large variations observed depending on the type of cancer (breast cancer 5.2% [1], rectal cancer 10% [2], gastric cancer 13.8% [3], liver cancer 21% [4], and oral cancer 40.6% [5]). Surgery for malignant musculoskeletal tumors is performed in the aseptic osteoarticular area. However, the incidence of SSI following surgery for this kind of tumor is anticipated to be higher than the incidence following orthopaedic surgery in general and the reasons for this include the following: (1) patients with malignant tumors require preoperative/postoperative chemotherapy and/or radiotherapy and (2) tumor resection creates a dead space. The onset of SSI following surgery for malignant musculoskeletal tumors can delay the start of postoperative adjuvant therapy, possibly leading to poor prognosis. To date, the incidence of SSI following orthopaedic surgery in general and the precautions for preventing SSI following such surgery have been studied sufficiently, yielding guidelines concerning the timing and duration of antimicrobial medication, techniques for operative field hair disposal, and so forth [6].

Malignant musculoskeletal tumors are relatively rare. Currently, there is no set of guidelines specific to the prevention of SSI following surgery for this kind of tumor because this issue has not yet been discussed sufficiently. The present study was undertaken to analyze data from patients with musculoskeletal tumors surgically treated at our department and to identify risk factors for SSI following surgery for malignant musculoskeletal tumors.

## 2. Patients and Methods

The study included 310 patients with benign musculoskeletal tumors and 147 patients with malignant musculoskeletal tumors who underwent surgery at our department between 2007 and 2012. Among these 147 patients, there were 22 metastatic tumor cases (14.9%). The incidence of SSI among these malignant musculoskeletal tumor patients was analyzed. According to the Centers for Disease Control and Prevention (CDC) definition, SSIs are classified as either incisional or of organ/space origin [7]. Incisional SSIs are further subclassified into those involving only skin and subcutaneous tissue (superficial incisional SSI) and those involving deeper soft tissues (deep incisional SSI). Organ/space SSIs involve any part of the anatomy (e.g., organ or space) other than incised body wall layers that were opened or manipulated during a surgical procedure [7].

In addition, factors related to the onset of SSI were analyzed in patients with malignant musculoskeletal tumors (*n* = 147). We analyzed factors affecting the incidence of infection: background variables (age, body mass index, presence/absence of hypertension, presence/absence of ischemic heart disease, presence/absence of diabetes mellitus, and presence/absence of preoperative chemotherapy) and surgery-related factors (skin incision size, duration of the surgery, blood loss, use of artificial materials, requirement of reconstructive surgery, and applicability of temporary wound closure). “Artificial materials” include prostheses, metal implants (screw, plates, and nails), and surgical meshes. “Reconstructive surgery” includes plastic surgical procedures such as musculocutaneous flap reconstruction, local skin flap reconstruction, and skin grafting. To analyze, in detail, the clinical courses of SSI cases, case-specific information such as isolated bacteria, treatment, oncological outcome, and treatment duration was collected. In addition, to explore biomarkers for the prediction and diagnosis of SSI, we analyzed hematological data (white blood cell [WBC] count, hemoglobin level [Hb], total protein level [TP], and C-reactive protein [CRP] level before operation; WBC, Hb, and TP on the day following operation; WBC and CRP one week after operation).

The standard protocols at our institution for prevention of SSI are based on the guidelines by the CDC [7] and the Society for Healthcare Epidemiology of America [8]. Briefly, these include reducing glycosylated hemoglobin A1c levels to 7% before surgery in diabetes patients, recommending smoking cessation within 30 days before the procedure, and improving nutritional status. The extrinsic procedure-related strategy includes no hair removal unless the hair that will interfere with the operation; if hair removal is necessary, it should be removed by clipping. The antimicrobial prophylaxis strategy includes administration within 1 hour before incision to maximize tissue concentration and withdrawal of prophylactic treatment within 24 hours after all procedures except cardiac surgery.

Statistical analysis was performed using Microsoft Excel. Statistical significance was analyzed using Student's *t*-test (one-tailed) or chi-square test, with *P* < 0.05 considered to indicate a significant difference. Odds ratio was used for analysis of risk factors.

To determine the musculoskeletal oncological surgery invasiveness (MOSI) index, 4 factors significantly associated with SSI development were chosen: operation duration, blood loss, preoperative chemotherapy, and use of artificial materials. To set a numerical cutoff for operation duration and blood loss, receiver operating characteristic (ROC) analysis was performed using Microsoft Excel. The point on the ROC curve closest to (0, 1) was selected as the optimal threshold (cutoff value) [9].

## 3. Results

The incidence of SSI was 0.4% (1/223) for cases of benign soft tissue tumors, 0% (0/87) for those of benign bone tumors, 11.8% (12/102) for those of malignant soft tissue tumors, and 13.0% (6/46) for those of malignant bone tumors, with the overall incidence of SSI being 12.2% for cases of malignant musculoskeletal tumors. Of the patients with malignant soft tissue tumors who developed SSI after surgery, 66.7% (8/12) had deep incision or organ/space SSI (Table 1). Among the patients with malignant bone tumor who developed SSI following surgery, 100% (6/6) had deep incision or organ/space SSI (Table 1).

Of the factors analyzed, ischemic heart disease (*P* = 0.001), preoperative chemotherapy (*P* = 0.003), skin incision length (*P* = 0.04), use of artificial materials (*P* = 0.01), duration of surgery (*P* = 0.0002), and blood loss (*P* = 0.0005) were significant risk factors for acquiring SSI (Table 2). Other factors analyzed in this study were not significantly associated with SSI (Table 2).

Results of the analysis of risk factor associations with SSI are presented in Table 2. The odds ratio (OR) was the highest for ischemic heart disease (OR: 10.4), followed by operation duration of ≥355 minutes (OR: 6.06), administration of preoperative chemotherapy (OR: 4.87), intraoperative blood loss of ≥190 g (OR: 4.39), and use of artificial materials (OR: 3.49).

Details of patients who developed SSI are presented in Table 3. The pathogens often identified were *Staphylococcus aureus *and coagulase-negative staphylococci. We also noted rare cases involving bacteria such as *Pseudomonas aeruginosa *and *Enterobacter *species as the pathogens of SSI. Of all cases of SSI, 7 cases (37%) required treatment for 1 year or longer, and 5 patients (28%) died after the onset of SSI (Table 3).

With regard to preoperative blood test data, the SSI and non-SSI groups did not differ significantly in terms of the WBC count, hemoglobin level, total protein level, or CRP level (Table 4). At 1 day after surgery as well, the WBC count and the hemoglobin and total protein levels did not differ significantly between the 2 groups (Table 4). With regard to the percent change at 1 day after surgery, the WBC count increased by 144% in the non-SSI group and by 154% in the SSI group relative to baseline values (preoperative level); however, these differences were not statistically significant (*P* = 0.44). The hemoglobin level decreased to 94% in the non-SSI group and to 88% in the SSI group relative to the baseline levels; these changes were not statistically significant either (*P* = 0.15). Further, the total protein level decreased to 82% in the non-SSI group and to 79% in the SSI group (*P* = 0.07). The levels of the 2 inflammation markers (WBC and CRP) at 1 week after surgery were significantly higher in the SSI group than in the non-SSI group (WBC *P* = 0.001 CRP *P* < 0.001) (Table 4).

ROC curve analysis revealed that the cutoff value for operation duration and blood loss was 355 minutes and 190 g, respectively. Therefore, the MOSI was calculated on the basis of each of these 4 factors (operation duration ≥355 m, blood loss ≥190 g, preoperative chemotherapy, and artificial material (Table 5)) using a 5-point scale (0–4). The average MOSI index of the SSI group (2.2 ± 0.3) was significantly higher than that of the non-SSI group (1.0 ± 0.1; *P* < 0.0001). The incidence of SSI was 38.5% when the MOSI index was 3-4 points and 7.1% at 0–2 points (Table 6).

## 4. Discussion 

Limb-sparing surgery is a currently common procedure for the treatment of malignant musculoskeletal tumors. However, because tumors often develop at sites that are anatomically difficult to treat (e.g., around major nerves and blood vessels), long operation times and high blood loss are common problems. Morii et al. reported that SSI developed in 7 (8.3%) of the 84 patients in their study after the surgical treatment of malignant soft tissue tumors, resulting in longer hospital stays [10]. In addition, they reported that intraoperative blood loss and tumor location (trunk) were significant risk factors for SSI and that the incidence of SSI did not differ according to age, tumor grade, use of preoperative chemotherapy, size of tumor, or the performance of accompanying plastic surgery [10]. For surgery in general, operation time [11, 12] and blood loss [4] have been reported as risk factors for SSI. These previous findings are consistent with the results of the present study. We considered that 4 factors (operation duration, blood loss, preoperative chemotherapy, and use of artificial materials) might reflect surgical invasiveness for the patients with malignant musculoskeletal tumors and would facilitate evaluation of the risk for SSI. However, operation duration and blood loss are sometimes correlated with each other. Therefore, we analyzed the statistical correlation of these 2 factors in our study. Pearson's correlation index was 0.542, which suggests that these 2 factors were not highly correlated in our study. One reason for this might be the difference between general orthopedic surgery and oncological surgery, in which we encounter massive blood loss in a short time period when dealing with hypervascular tumors. To test our hypothesis, the relationship between the MOSI index and the incidence of SSI was analyzed. As shown in Table 6, the MOSI index was significantly correlated with the incidence of SSI (*P* < 0.0005). These results suggest that the risk for SSI onset can be predicted to be very high (OR 8.82) in cases in which the MOSI index based on preoperatively estimated blood loss and operation time, and so forth, is 3 points or higher. A further study involving a larger number of patients is needed to verify the usefulness and validity of this index.

As a preoperative risk factor for SSI, preoperative chemotherapy was shown to elevate the incidence of SSI in a slight but statistically significant manner, suggesting that this factor affects the immune potentials of patients undergoing surgery. We analyzed blood data to determine the preoperative and postoperative condition (including immune function) of individual patients. None of the preoperative blood parameters analyzed was identified as a predictive factor for SSI. We hypothesized that the blood parameter data at 1 day after surgery would reflect the effects of surgery (bleeding, dehydration, inflammation, and malnutrition), possibly enabling prediction of SSI. In fact, however, there was no significant difference between the SSI group and the non-SSI group in terms of the WBC count, hemoglobin level, or total protein level. Next, we analyzed the differences in the percent changes in these 3 parameters at preoperative baseline and at 1 day after surgery. This analysis revealed a larger percent change in total protein levels in the SSI group (21% decrease) than in non-SSI group (18% decrease), but the difference was not significant (*P* = 0.07) possibly because of the limited number of subjects. Although blood loss was identified as a significant factor, postoperative hemoglobin levels did not differ between the 2 groups. This seems to reflect the influences of dehydration and blood transfusion.

Standard measures at our facility for the prevention of SSI include strict blood glucose control for diabetic patients and the use of antimicrobial agents (cephalosporins) before surgery until the day after surgery [7, 8]. The results of the present study indicate that the new measures to be adopted for the prevention of SSI should be careful observation of clinical symptoms (e.g., postoperative fever and local findings) and frequent blood tests in cases with an MOSI index of more than 2, so that early detection of SSI can be facilitated. It might be worthwhile to reconsider the use of antimicrobial agents in high-risk patients. Routine use of vancomycin is not recommended to prevent emergence of vancomycin-resistant microbials [7, 8]. However, the Society for Healthcare Epidemiology of America recommends the use of vancomycin in high-risk surgical procedures especially in procedures requiring the placement of implants [8]. Therefore, we could consider using vancomycin as an antimicrobial prophylactic agent in patients with MST who are at high risk for SSI.

Furthermore, in cases of patients with a high CRP level and WBC count at 1 week after surgery, SSI should be strongly suspected. In such cases, it is advisable to take preemptive measures such as performing diagnostic imaging studies (e.g., ultrasonography and computed tomography) and prompt exploration of the wound (puncture or opening). When dealing with patients with an elevated risk for SSI, we often administer hyperbaric oxygen therapy (HBOT). HBOT is considered to contribute to wound healing by stimulating neovascularization and oxygen tissue diffusion and thus improving oxygen transport to the wound [13]. The effects of HBOT on infection are based on several mechanisms, including reinforcement of the bacterial killing capacity of neutrophils [14], alleviation of inflammation and edema [15], and reinforcement of antimicrobial drug efficacy [16] (reviewed by Hopf and Holm [17]). We will clarify the effects of HBOT in the prevention and treatment of SSI in future separate studies. When performing surgery for musculoskeletal tumors for which no standard surgical-anatomical approach has been defined, the operation plan should be carefully drafted with discussion in preoperative case conferences, with goals set at shortening the operation time and reducing blood loss using measures specific to this kind of surgery. To this end, acquisition of good operative skill is needed, such as anatomical approach, handling of the vessels, or procedures of implant surgery. It may also be desirable to perform reconstruction simultaneously with tumor resection. As one such attempt, we have begun to incorporate preoperative simulation with a 3D-printer-created tumor model [18] and surgical training using the model. Preoperative embolization has been reported as a measure to be taken for hypervascular metastatic bone tumors [19] and it has also been reported to reduce blood loss when applied before surgery for sarcoma [20]. In our department, for some cases, embolization is performed on the basis of findings from angiograms taken on the day before surgery [21].

Although the use of artificial materials has been identified as a risk factor for infection, their use in surgery for malignant musculoskeletal tumors is often unavoidable. Gosheger et al. reported that failure due to infection occurred in 30 (12%) of the 250 patients undergoing musculoskeletal sarcoma resection and prosthetic joint reconstruction, thus demonstrating an incidence of failure higher than that associated with TKA and THA in general [22]. Some investigators reported that if the tumor-type joint prosthesis is positively combined with a flap, the postoperative complications can be reduced and the limb preservation rate can be increased [23]. Reconstructive surgery involving plastic surgery techniques is indispensable as a countermeasure for the dead cavity created after resection of giant soft tissue tumors [24]. We also use musculocutaneous flaps such as a rectus abdominis musculocutaneous flap, a sural musculocutaneous flap, and a latissimus dorsi musculocutaneous flap, sartorius to reconstruct the tissue defects. However, in the present study, the onset of SSI remained unaffected by the performance of plastic reconstructive surgery aimed at improving the coverage of the artificial materials and preventing the failure of wound healing. Prolonged duration of surgery resulting from adoption of complex reconstructive procedures is a dilemma we may continue to face. One possible option, which deserves discussion, may be to perform reconstruction as a two-stage operation so that the operation time can be shortened.

In the analysis of pathogens, *Staphylococcus* was isolated from 14 of the 18 cases, consistent with a past report [6, 10]. Methicillin-resistant *Staphylococcus aureus *(MRSA) was responsible for infection in 6 cases, including 3 cases in which the control of infection was not possible and the patient died without receiving appropriate postoperative chemotherapy (Table 4). Rao et al. reported that preoperative screening for *Staphylococcus aureus* within the nasal cavity and its eradication can reduce the incidence of *Staphylococcus aureus *SSI in patients undergoing orthopaedic surgery [25]. Similarly, at our facility, we make it a rule to perform bacterial screening of the nasal cavity in all cases and to perform bacterial eradication with mupirocin ointment in MRSA-positive cases. This practice is supported by a publication recommending the use of anti-MRSA drugs at the time of surgery instead of cefem family antibiotics for MRSA-positive patients [6]. In the present study, none of the 7 patients that developed SSI due to MRSA had been MRSA-positive preoperatively, suggesting that onset of SSI through endogenous MRSA infection was prevented in the present study. However, the fact that many patients developed SSI due to MRSA suggests that infection was due to MRSA transmission via healthcare workers or from the environment. It therefore seems necessary to review the current measures taken for the prevention of perioperative infection, including compliance with standard preventive measures (ensuring hand/finger cleanliness among healthcare workers), compliance with measures for the prevention of infection through contact with MRSA-positive patients, and appropriate use of antibacterial drugs to avoid selection of drug-resistant bacteria. This is particularly important when caring for patients with musculoskeletal tumors, which require more intense physical care than usual. In another recent study, we analyzed the MRSA genotype and biofilm-forming capability. We found that the biofilm-forming capability was increased in MRSA strains isolated from patients with SSI following surgery with the use of artificial materials [26]. In addition, the presence of the *agr*-2 gene was associated with biofilm-forming capability, indicating that biofilm-forming capability can be quickly evaluated by assaying for this gene. This is a potentially useful tool for the treatment and targeting of biofilms.

## 5. Conclusion

Blood loss, duration of surgery, skin incision size, and use of artificial materials were identified as risk factors associated with the onset of SSI after surgery for musculoskeletal tumors. Patient risk factors for SSI were preoperative chemotherapy and ischemic heart disease. Careful observation and early detection/treatment of SSI on the basis of the risk for SSI (estimated by the MOSI index) and inflammatory reactions at 1 week after surgery are important as countermeasures against SSI following surgery for musculoskeletal tumors, which can result in death as the worst outcome.

## Figures and Tables

**Table 1:** Incidence of SSI by tumor types.

	SSI	Deep/organ SSI
Benign tumor		
Benign bone tumor (*n* = 87)	0	
Benign soft tissue tumor (*n* = 223)	1 (0.4%)	1 (100%)
Malignant tumor		
Malignant bone tumor (*n* = 46)	6 (13.0%)	6 (100%)
Malignant soft tissue tumor (*n* = 102)	12 (11.8%)	8 (66.7%)

**Table 2:** Analysis of risk factors for surgical site infection in malignant bone and soft tissue tumors.

	Non-SSI (*n* = 129)	SSI (*n* = 18)	Odds ratio	95% CI	*P* value
Age	57.6 ± 18.4	58.7 ± 17.6			0.40
Aged case (>60 y)	73 (53.3%)	12 (66.7%)	1.62	0.57–4.57	0.36
Gender (male/female)	66/63	6/12	0.48	0.16–1.35	0.16
BMI (kg/m^2^)	23.0 ± 3.8	23.8 ± 3.1			0.20
Overweight (>25)	40 (31.0%)	7 (38.9%)	1.41	0.51–3.92	0.50
Hypertension	32 (25%)	6 (33.0%)	1.51	0.53–4.37	0.44
Ischemic heart disease	4 (3.1%)	4 (22.2%)	10.4	2.31–47.0	0.001
Diabetes	15 (11.6%)	2 (11.1%)	0.95	0.19–4.54	0.95
Tumor location (Trunk/Extremity)	14/111	4/14	1.76	0.52–5.95	0.36
Primary/metastatic tumor	110/19	15/3	1.15	0.30–4.38	0.99
Preoperative chemotherapy	12 (9.3%)	6 (33.3%)	4.87	1.55–15.3	0.003
Skin incision (cm)	21.3 ± 12.0	23.1 ± 9.3			0.04
Large skin incision (>25 cm)	41 (31.8%)	10 (55.6%)	2.68	0.68–5.0	0.047
Use of artificial materials	34 (26.4%)	10 (55.6%)	3.49	1.27–9.58	0.01
Reconstructive procedure	42 (32.6%)	6 (33.3%)	1.04	0.37–2.95	0.95
Secondary wound closure	25 (19.4%)	3 (16.7%)	0.83	0.22–3.10	0.78
Duration of surgery (min)	265 ± 155	413 ± 202			0.0002
Prolonged surgery (≥355 min)	32 (24.8%)	12 (66.7%)	6.06	2.10–17.4	0.0003
Blood loss (g)	270 ± 431	726 ± 1053			0.0005
Massive blood loss (≥190 g)	23 (17.4%)	9 (50.0%)	4.39	1.47–13.0	0.005

**Table 3:** Case-specific data of SSI patients of malignant musculoskeletal tumors.

	Age/sex	Diagnosis	Site	Surgical procedure	Reconstruction	Duration of surgery (min)	Blood loss (g)	Isolated bacteria from SSI	Treatment for SSI	Oncological outcome/SSI healing	Treatment duration for SSI (month)
1	62/F	Metastatic bone tumor	Sacrum	Marginal resection	Artificial mesh	355	978	MRSA	Antibiotics, HBOT, surgery	DOD/not healed	18
2	61/M	Chondrosarcoma	Femur	Wide resection	Pasteurized autograft, plate and screw	383	500	CNS	Antibiotics, HBOT, surgery	CDF/healed	12
3	62/M	Metastatic bone tumor (thyroid)	Humerus	Marginal resection	Tumor-prosthesis	397	270	MSSA	Antibiotics, HBOT, surgery	DOD/healed	5
4	13/M	Osteosarcoma	Tibia	Wide resection	Tumor-prosthesis Gastrocnemius muscle flap	445	470	MRSA	Antibiotics, HBOT, surgery	CDF/healed	9
5	35/M	Osteosarcoma	Pelvis	Hemipelvectomy	Liquid-nitrogen treated autograft, plate and screw, external fixator	890	4090	CNS	Antibiotics, HBOT, surgery	DOD/not healed	12
6	61/M	Metastatic bone tumor (kidney)	Femur	Wide resection	Tumor-prosthesis, Sartorius muscle flap	514	600	MSSA	Antibiotics, surgery (amputation)	AWD/healed	1
7	45/M	Myxoid liposarcoma	Thigh	Wide resection including Femur	Pasteurized autograft, plate and screw	680	1620	MRSA	Antibiotics, HBOT, surgery	DOD/not healed	18
8	79/F	MFH	Buttock	Wide resection	Gluteal artery perforator flap	145	50	MRSA	Antibiotics, HBOT	DOO/not healed	9
9	64/M	MFH	Thigh	Marginal resection	None	508	2505	*Pseudomonas aeruginosa *	Antibiotics, HBOT, surgery	DOD/not healed	9
10	82/M	Dedifferentiated liposarcoma	Thigh	Disarticulation of hip	None	391	220	*Enterobacter cloacae *	Antibiotics, surgery	CDF/healed	5
11	45/M	Myxoid liposarcoma	Thigh	Wide resection including Femur	Pasteurized autograft, plate and screw	550	470	CNS	Antibiotics, HBOT, surgery	CDF/healed	18
12	75/M	MFH	Lower leg	Wide resection including Tibia	Bone cement, intramedullary nail, Gastrocnemius muscle flap, FTSG	283	255	CNS	Antibiotics, HBOT, surgery (local flap)	CDF/not healed	21
13	30/M	MFH	Groin	Wide resection	None	250	105	*Pseudomonas aeruginosa *	Antibiotics, HBOT, surgery (rectus abdominis muscle flap)	CDF/not healed	3
14	70/F	MFH	Thigh	Marginal resection	Gastrocnemius muscle flap, FTSG	163	30	MSSA	Antibiotics, HBOT, NPWT	CDF/healed	3
15	59/M	MFH	Thigh	Wide resection	None	146	0	MSSA	Antibiotics, HBOT,	AWD/healed	1
16	73/F	Chondrosarcoma	Hand	Marginal resection including metatarsal bone	Iliac autograft, Kirschner wire	524	685	*Enterobacter cloacae *	Antibiotics, surgery (forearm flap)	CDF/healed	20
17	73/F	MFH	Thigh	Wide resection	Free latissimus dorsi flap, STSG	626	190	MRSA	Antibiotics, HBOT, surgery, NPWT	AWD/healed	3
18	68/F	Undifferentiated sarcoma	Thigh	Wide resection	STSG	189	30	MRSA	Antibiotics	CDF/healed	2

MFH: malignant fibrous histiocytoma (pleomorphic undifferentiated sarcoma); FTSG: full-thickness skin graft; STSG: split-thickness skin graft; MSSA: methicillin-sensitive *Staphylococcus aureus*; mRSA: Methicillin-resistant *Staphylococcus aureus*; CNS: coagulase-negative staphylococci; HBOT: hyperbaric oxygen therapy; NPWT: negative-pressure wound therapy; CDF: continuous disease free; DOD: died of disease; DOO: died of other cause (Case 8, uterine cervical cancer); AWD: alive with disease.

**Table 4:** Analysis of pre- and postoperative laboratory values.

	Non-SSI	SSI	*P* value
Preoperative values			
WBC (/m^3^)	6,242 ± 278	6,016 ± 405	0.41
Hemoglobin (g/dL)	12.3 ± 0.2	12.3 ± 0.5	0.49
Total protein (g/dL)	6.9 ± 0.1	7.0 ± 0.1	0.32
CRP (mg/dL)	1.6 ± 0.3	1.4 ± 0.5	0.41
Postoperative (1 day) values			
WBC (/m^3^)	8,959 ± 285	9,245 ± 602	0.36
Hemoglobin (g/dL)	11.3 ± 0.17	10.8 ± 0.4	0.17
Total protein (g/dL)	5.7 ± 0.7	5.5 ± 0.2	0.12
Postoperative (1 week) values			
WBC (/m^3^)	6,528 ± 230	8,689 ± 993	0.001
CRP (mg/dL)	2.2 ± 0.3	8.8 ± 2.1	<0.0001

**Table 5:** Musculoskeletal oncological surgery invasiveness index (MOSI index).

Value	Points
Duration of surgery (min)	
<355	0
≥355	1
Blood loss (g)	
<190	0
≥190	1
Preoperative chemotherapy	
No	0
Yes	1
Artificial materials	
No	0
Yes	1

**Table 6:** Relationship between the incidence of SSI and the musculoskeletal oncological surgery invasiveness (MOSI) index.

MOSI index (points)	SSI (%)
3-4	38.5*
0–2	7.1

*P < 0.0005 versus cases of 0–2 points.
